# Comparison of the effectiveness and safety of perampanel and oxcarbazepine as monotherapy in children and adolescents with newly diagnosed focal epilepsy

**DOI:** 10.3389/fphar.2023.1189058

**Published:** 2023-08-30

**Authors:** Jia-Qin Yi, Sheng Huang, Miao-Juan Wu, Jie-Hui Ma, Li-Juan Huang, Song Liang, Dan Sun

**Affiliations:** ^1^ Department of Neurology, Wuhan Children’s Hospital of Tongji Medical College, Huazhong University of Science and Technology, Wuhan, China; ^2^ Department of Pediatric Rehabilitation, Hubei the Third People’s Hospital, Wuhan, China

**Keywords:** perampanel, oxcarbazepine, newly diagnosed focal epilepsy, monotherapy, anti-seizure medications

## Abstract

**Objective:** This study aims to compare the effectiveness and safety of perampanel and oxcarbazepine as monotherapy in children with focal epilepsy (FE).

**Methods:** This is an ambispective, single-center, non-inferiority study comparing the effectiveness and safety of perampanel (PER) monotherapy and oxcarbazepine (OXC) monotherapy in children with newly diagnosed FE. The primary endpoint was a six-month seizure freedom rate. The secondary endpoints included retention, responder, and seizure freedom rates at 3, 6, and 12 months, respectively. Adverse events (AEs) were also recorded for both groups.

**Results:** One hundred and thirty children and adolescents aged from 4 to 18years newly diagnosed with FE between May 2020 and November 2022 in Wuhan Children’s Hospital were included. There were 71 patients in the PER group and 59 patients in the OXC group. In the per protocol set (PPS), 50 (78.1%) in the PER group and 43 (78.2%) in the OXC group completed six months of treatment without seizures. The lower 95% CI (66.0%–87.5%) limit of PER was higher than the non-inferiority margin of 62.4% (80% of the 6-month seizure freedom rate in the OXC group); PER was non-inferior to OXC. The 3-month and 12-month seizure freedom rates were 77.1% and 82.9% for the PER group, respectively, while they were 80.4% and 75.8% for the OXC group. There were no serious adverse events in both groups.

**Conclusion:** PER showed comparable effectiveness and safety compared with OXC in children with newly diagnosed focal epilepsy, which might be an effective and safe treatment for children and adolescents with newly diagnosed FE.

**Clinical Trial Registration:** Identifier ChiCTR2300074696

## 1 Introduction

Epilepsy is a chronic neurologic disorder affecting over 70 million people worldwide, especially children (Löscher et al., 2020). Compared with generalized epilepsy, focal epilepsy (FE) is more in common both children and adults (Beghi, 2020). Approximately 60%–70% of people with epilepsy (PWE) will achieve long-term remission after starting anti-seizure medications (ASMs), and most PWE are treated with monotherapy (Nevitt et al., 2022). Therefore, the selection of an effective ASM for initial monotherapy remains a critical and challenging process, especially in children.

Oxcarbazepine (OXC) has been considered the first-choice for the initial treatment of FE due to its efficacy and tolerability (Kanner and Bicchi, 2022). However, a range of new ASMs are available, and evidence on their effectiveness and tolerability for FE is needed (Abou-Khalil, 2022; Nevitt et al., 2022). Perampanel (PER) is a third-generation ASM, which acts as a selective, non-competitive α-amnio-3-hydroxy-methyl-4-isoxazolepropionic acid (AMPA) receptor antagonist to reduce glutamate-mediated postsynaptic excitation. PER is approved for use as adjunctive therapy and monotherapy in the treatment of FE with or without focal to bilateral tonic–clonic seizures (FBTCSs) in patients aged 4 years and older (Kanner et al., 2018). The efficacy, safety, and tolerability profiles of adjunctive PER in pediatric patients with FE with or without FBTCSs have been well demonstrated in randomized controlled trials and real-world studies (Lin et al., 2018; Fogarasi et al., 2020; Hwang et al., 2020; Piña-Garza et al., 2020). Moreover, another observational study showed its good efficacy and tolerability in association with 1–2 ASMs without using a high dose in both pediatric and adult patients during up to 24-month follow-up (Macrohon et al., 2021). However, there are limited data regarding PER used as monotherapy in pediatric patients, and evidence directly comparing PER monotherapy with other ASMs is also lacking. A few cross-sectional descriptive and retrospective studies have reported preliminary experience with a limited number of pediatric patients initially treated with PER as monotherapy (Toledano Delgado et al., 2020; Macrohon et al., 2021; Li et al., 2022). Additionally, a real-life experience demonstrated that PER monotherapy seems effective and well tolerated in 20 patients, aged between 8 and 10, with childhood absence seizures (Operto et al., 2020b; Operto et al., 2022). Furthermore, clinical experience with PER monotherapy has mainly been addressed in adult patients (Yamamoto et al., 2020; Chinvarun, 2022; Wechsler et al., 2022). Efficacy in adults can be extrapolated to children aged 4 years and older.

Therefore, the aim of this study was to compare the effectiveness and safety of OXC and PER in pediatric patients from real-world experience, which could further guide the selection of ASM monotherapy in the pediatric population.

## 2 Methods

### 2.1 Study population

This is an ambispective cohort study. Retrospective data were collected from May 2020 to June 2022, and all the included children and adolescents were prospectively followed until November 2022.

We reviewed clinical data of children and adolescents with newly diagnosed FE who started PER or OXC as the first ASM monotherapy at Wuhan Children’s Hospital. Eligible participants included children and adolescents aged 4–18 years with newly diagnosed FE with or without FBTCSs according to the 2017 ILAE (Fisher et al., 2017) Classification of Epileptic Seizures who had clinical or electroencephalographic (EEG) findings suggesting FE. Furthermore, children and adolescents should have experienced at least two unprovoked seizures, within the previous 3 months, with a minimum separation of 24 h between the seizures, and one of these seizures should have occurred within the last 1 month. In addition, an EEG and a brain CT or MRI examination were also needed. The exclusion criteria included 1) children and adolescents who experienced generalized seizures or had generalized spike-wave discharges verified by EEG; 2) children and adolescents who had used other ASMs (except for those used as rescue treatment within 4 weeks prior to the study).

This study has been approved by the Ethics Committee of Wuhan Children’s Hospital.

### 2.2 Treatment

The enrolled children and adolescents received an initial dose of 1–2 mg/day before bedtime or 5–10 mg/kg/day of oxcarbazepine as one dose in the morning and one in the evening, around 12 h apart. If no tolerability issues occurred, PER would be up-titrated to 4 mg/d and OXC to 10–20 mg/kg/d as the minimal maintenance dosage for 2 weeks, respectively.

If children who tolerated PER at a dose of 4 mg/d or OXC at a dose of 10–20 mg/kg/d reported seizures during the follow-up period, the dose of PER or OXC will be up-titrated to the higher level (PER maximally to 8 mg/d and OXC maximally to 30–40 mg/kg/d). Down titration was allowed when children and adolescents experienced intolerable adverse events. If patients could not tolerate adverse events or had uncontrolled seizures after dose adjustment, PER or OXC could be switched to alternative monotherapy or polytherapy. The enrolled patients were not treated with other non-pharmacological treatments in progress.

### 2.3 Efficacy and safety endpoints

The primary outcome was the seizure freedom rate, which was defined as the proportion of children and adolescents remaining seizure-free at 6 months. The secondary outcome was the retention rate of PER and OXC at 3, 6, and 12 months, respectively. Other efficacy outcomes included the 50% responder rate, which was defined as the proportion of children and adolescents achieving a ≥50% reduction in seizure frequency compared with baseline, 75% responder rate (the proportion of children and adolescents achieving a ≥75% reduction in seizure frequency compared with baseline), the seizure freedom rate at different visit points, the time to the first seizure onset (defined as the period from the first perampanel dose to the occurrence of the first seizure), and the time to treatment failure (defined as withdrawal from the study due to adverse events or lack of efficacy).

The clinical data, including age, age category, sex, weight, time since diagnosis, baseline seizure frequency, seizure type, epileptic syndrome, etiology, and intellectual disability, were also collected. Adverse events (AEs) and serious AEs were recorded and accessed in the full analysis set (FAS), which was defined as children and adolescents who received at least one dose of the trial treatment.

### 2.4 Statistical analysis

The primary outcome was analyzed in the per protocol set (PPS). The per protocol set included children in FAS and had no important deviations that could affect the primary outcome (missing seizure data and poor treatment compliance). It was assumed that an acceptable lower cutoff value was determined by calculating 80% of the comparator’s (herein OXC) six-month seizure freedom rate (Glauser et al., 2013). Once this lowest acceptable cutoff was established, the 95% lower confidence limit of the six-month seizure freedom rate of the PER group was calculated in the PPS. PER monotherapy treatment was considered non-inferior to OXC if the 95% lower confidence limit was above the lower acceptable cutoff.

Kalpan–Meier methods were used to calculate the time to the first seizure onset and the time to withdrawal from the study. Adverse events were summarized for the full analysis set. The statistical analysis was performed using the Statistical Analysis System 9.4 Institute, Cary, NC, United States. Descriptive data were expressed as the mean and standard deviation (SD). In case of a non-normal distributed variable, the median and inter quartile range (IQR) were shown. Categorical variables were expressed as frequency and percentage. To compare continuous variables, analysis of variance and the Wilcoxon test were used. For categorical data, Pearson’s Chi-square, Yate’s correction for continuity, and Fisher’s exact test were used. *p*-value was set at *p* <0.05 for statistical significance.

## 3 Results

### 3.1 Basic characteristics

**FIGURE 1:**
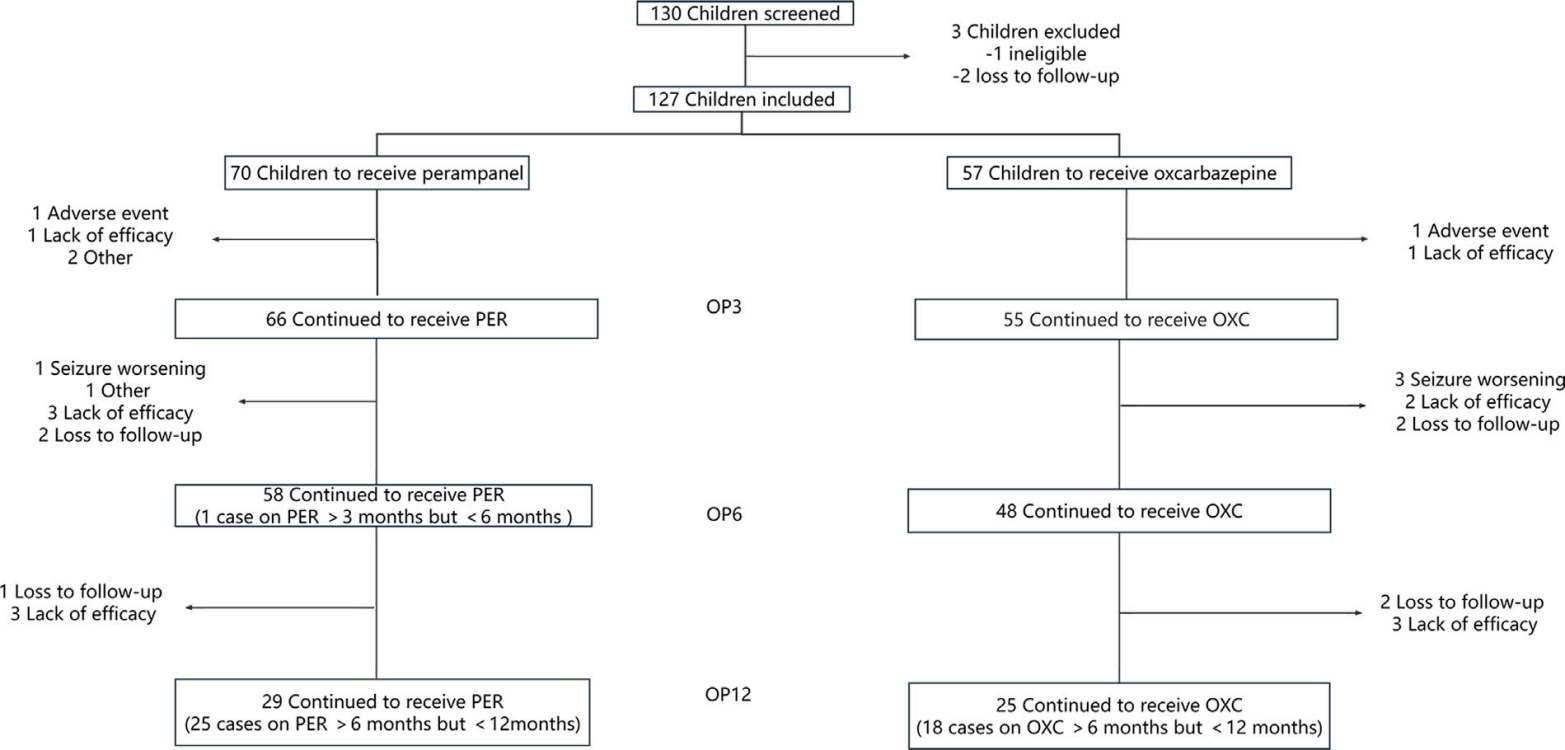
Illustration of the number of children evaluated at each observation point (OP) who have been treated with perampanel (PER) or oxcarbazepine (OXC) monotherapy. OP3: observation point at 3 months; OP6: observation point at 6 months; and OP12: observation point at 12 months.

A total of 130 children and adolescents from Wuhan Children’s Hospital were screened in the study. One hundred and twenty-seven children and adolescents were included in the full-set analysis, while there were 70 patients in the PER group and 57 in the OXC group. The patient flow chart is shown in Figure 1.

The basic characteristics are shown in Table 1. Compared with the PER group, children and adolescents in the OXC group started treatment earlier after seizure onset (12.99 ± 23.28 months in the PER group versus 6.89 ± 10.44 months in the OXC group, *p* = 0.028). The proportion of children and adolescents with a seizure frequency >10 per month in the OXC group was higher than that in the PER group (10.5% vs. 1.4%, *p* = 0.025).

**TABLE 1:** Patients’ demographics and baseline clinical characteristics.

Characteristics	Perampanel (*n* = 70)	Oxcarbazepine (*n* = 57)	*p*-value
Age (years), mean ± SD	8.6 ± 2.5	8.1 ± 2.9	0.207
Age category, n (%)			0.716
≥4 and ≤7 years	26 (37.1%)	25 (43.9%)	
>7 and ≤12 years	36 (51.4%)	27 (47.4%)	
>12 years	8 (11.4%)	5 (8.8%)	
Sex, n (%)			0.942
Male	45 (64.3%)	37 (64.9%)	
Female	25 (35.7%)	20 (35.1%)	
Weight (kg), mean ± SD	33.2 ± 15.3	29.0 ± 12.1	0.116
Time since diagnosis (months), mean ± SD	13.0 ± 23.3	6.9 ± 10.4	0.028
Monthly seizure frequency, mean ± SD (minimum, maximum)	3.0 ± 2.2 (1.0, 13.0)	3.6 ± 4.2 (1.0, 24.0)	0.645
Monthly seizure frequency, n (%)			0.025
>10 seizures	1 (1.4%)	6 (10.5%)	
≤10 seizures	69 (98.6%)	51 (89.5%)	
Seizure type, n (%)			
FAS	4 (5.7%)	4 (7.0%)	>0.999
FIAS	38 (54.3%)	23 (40.4%)	0.118
FBTCS	28 (40.0%)	30 (52.6%)	0.155
Epileptic syndrome, n (%)			0.059
BECT	23 (32.9%)	16 (28.1%)	
ESES	2 (2.9%)	0	
TSC	0	3 (5.3%)	
OLE	1 (1.4%)	0	
FLE	0	4 (7.0%)	
Not classified as epileptic syndrome	44 (62.9%)	34 (59.6%)	
Etiology, n (%)			0.230
Genetics	5 (7.1%)	4 (7.0%)	
Structural	13 (18.6%)	4 (7.0%)	
Metabolic	1 (1.4%)	0	
Unknown	51 (72.8%)	49 (86.0%)	
Intellectual disability, n (%)	11 (15.7%)	9 (15.8%)	0.650

FASs, focal awareness seizures; FIASs, focal-impaired awareness seizures; FBTCSs, focal to bilateral tonic–clonic seizures; BECT, benign childhood epilepsy with centrotemporal spikes; ESES, electrical status epilepticus during sleep; TSC, tuberous sclerosis complex; OLE, occipital lobe epilepsy; FLE, frontal lobe epilepsy; SD, standard deviation.

### 3.2 Primary endpoint

The duration of treatment between the two groups was similar. The median PER maintenance dosage was 4 mg (range 1–6 mg), and the OXC dosage was 22.70 mg/kg/d (range 13.95–35.29 mg/kg/day). In the PPS, 78.1% (50/64, 95% CI: 66.0%–87.5%) of children in the PER group and 78.2% (43/55) in the OXC group were seizure-free at 6 months. The lower limit of the 95% CI in the PER group was above the non-inferiority limit set (62.4%, 80% of the 6-month seizure freedom rate in the OXC group); therefore, PER was non-inferior to OXC. PER was also non-inferior to OXC in FAS analysis (Table 2).

**TABLE 2:** Six-month seizure freedom proportion in the PPS and FAS population.

	PPS		FAS	
	Perampanel	Oxcarbazepine	Perampanel	Oxcarbazepine
6-month seizure freedom rates	78.1%	78.2%	72.5%	75.4%
80% of OXC 6-month seizure freedom rates	—	62.4%	—	60.3%
95% CI	66.0%–87.5%	—	60.4%–82.5%	—

PPS, per protocol set; FAS, full analysis set; CI, confidence interval; OXC, oxcarbazepine.

### 3.3 Secondary endpoints

**FIGURE 2:**
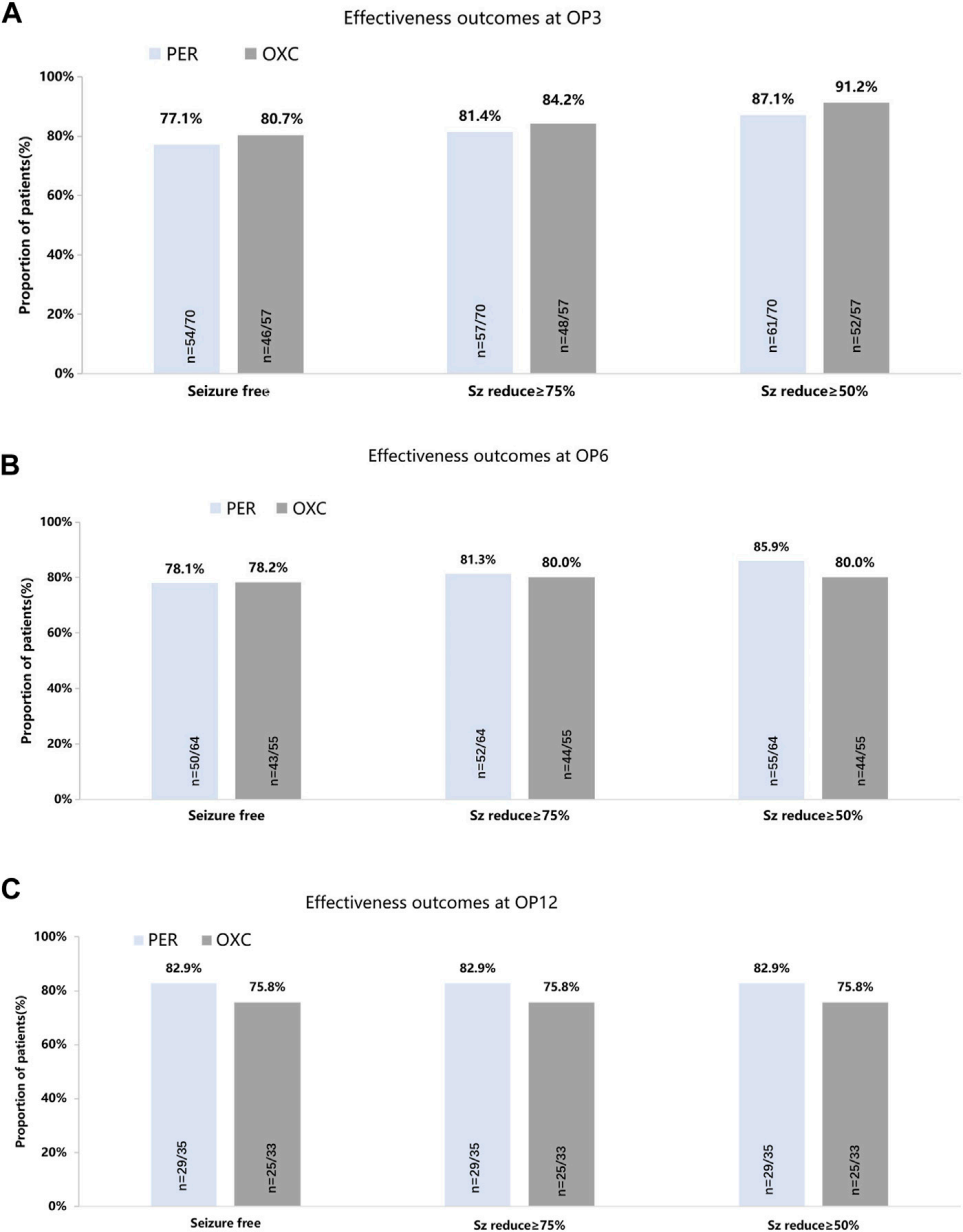
Seizure-response status and seizure-free status on PER/OXC monotherapy. **(A)** OP3; **(B)** OP6; and **(C)** OP12. Seizure free: seizure freedom; Sz reduce ≥75%: seizure reduction ≥75%; Sz reduce ≥50%: seizure reduction ≥50%.

**FIGURE 3:**
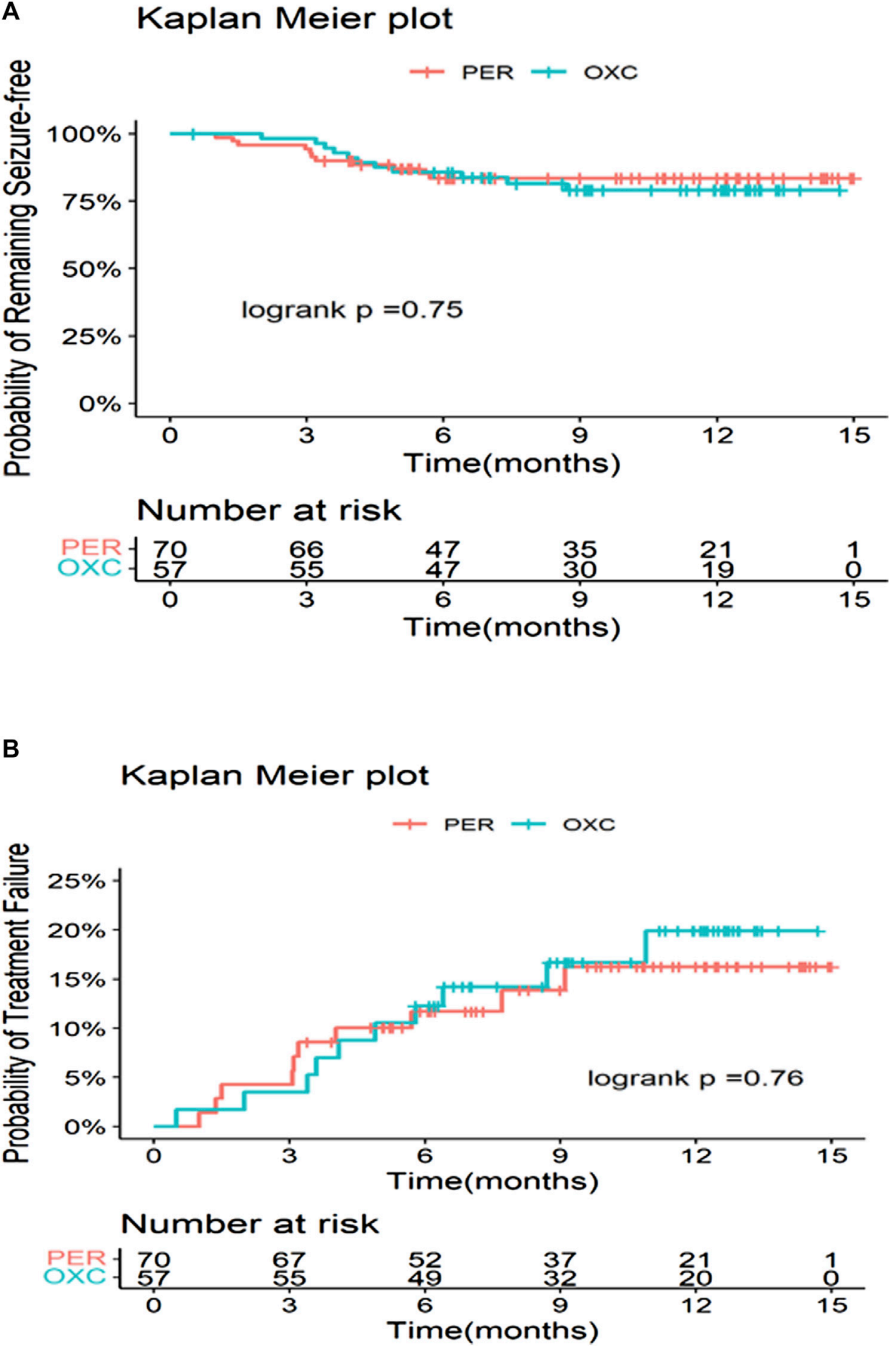
Time to first seizure and treatment failure in the PER **(A)** and OXC **(B)** groups.

The seizure freedom, seizure reduction ≥75%, and seizure reduction ≥50% showed no significant difference between the PER and OXC groups at 3, 6, and 12 months (*p* > 0.05) (Figure 2). Treatment failure was the most common reason for discontinuation in the PER group (seven cases) and the OXC group (six cases). At 3 months, the retention rates of the PER and OXC groups were 94.3% (66/70) and 96.5% (55/57), respectively (*p* = 0.5601). The retention rate at 6 months was 84.1% (58/70) and 84.2% (48/57) for the PER and OXC groups, respectively (*p* = 0.9814). The retention rate at 12 months was 78.4% (29/38) and 69.4% (25/36) for the PER and OXC groups, respectively (*p* = 0.3845). There were no significant differences in the time to the first seizure onset and treatment failure between the two groups (*p* > 0.05) (Figure 3). The probabilities of children and adolescents remaining seizure-free with PER and OXC were 83.5% (95% CI: 72.0%, 90.5%) and 85.7% (95% CI: 73.5%, 92.6%) at 6 months, respectively.

### 3.4 Tolerability and safety

There were 15 (21.4%) and 16 (28.1%) AEs in the PER and OXC groups, respectively. There was no serious AE in both groups. The incidence of AEs requiring a dose reduction or discontinuation was 6% and 2% in the PER groups and 1% and 3% in the OXC group. The most common AEs in the PER group were dizziness (five cases), somnolence (three cases), irritability (three cases), and restless sleep (two cases). In the OXC group, the most common AEs were rash (five cases), restless sleep (two cases), seizure worsening (two cases), nausea (two cases), and abnormal laboratory parameters (two cases) (Table 3).

**TABLE 3:** Summary of AEs.

	Perampanel (n = 70)	Oxcarbazepine (n = 57)	*p*-value	
Patients with any AEs, n (%)	15 (21.1%)	16 (28.1%)	0.386	
Serious AEs, n (%)	0 (0.0%)	0 (0.0%)	—	
AEs leading to discontinuation/dose adjustment			0.080	
Discontinuation, n (%)	2 (2.9%)	3 (5.3%)		
Dose increase, n (%)	1 (1.4%)	0 (0.0%)		
Dose reduction, n (%)	6 (8.6%)	1 (1.8%)		
No adjustment, n (%)	6 (8.6%)	12 (21.1%)		
AEs				
Rash, n (%)	0 (0.0%)	5 (8.8%)	0.039	
Somnolence, n (%)	3 (4.3%)	0 (0.0%)	0.320	
Irritability, n (%)	3 (4.3%)	0 (0.0%)	0.320	
Restless sleep, n (%)	2 (2.9%)	2 (3.5%)	0.764	
Dizziness, n (%)	5 (7.1%)	1 (1.8%)	0.315	
Abnormal behavior, n (%)	0 (0.0%)	1 (1.8%)	0.920	
Increased weight, n (%)	1 (1.4%)	0 (0.0%)	0.920	
Seizure worsening, n (%)	1 (1.4%)	2 (3.5%)	0.863	
Nausea, n (%)	0 (0.0%)	2 (3.5%)	0.387	
Abnormal laboratory parameters, n (%)	0 (0.0%)	2^a^(3.5%)	0.387	
Memory deterioration, n (%)	0 (0.0%)	1 (1.8%)	0.920	

AE, adverse event.

^a^ One child with serum electrolyte changes and the other with elevated serum myocardial enzymes.

## 4 Discussion

The current study investigated the effectiveness and safety of PER and OXC as monotherapy in children and adolescents with FE with or without FBTCSs. The effectiveness and safety of PER and OXC were similar. PER was non-inferior to OXC in terms of seizure freedom rates after 6 months of treatment. PER may be expected as the first choice for the treatment of FE with or without FBTCSs in children and adolescents.

ILAE evidence review reported that OXC was established as the only adequate comparator as initial monotherapy for children with newly diagnosed FE (Glauser et al., 2013). While previous studies have demonstrated similar effectiveness and good tolerability of PER monotherapy, there is limited clinical evidence regarding its use in children, particularly the lack of head-to-head comparisons with other ASMs. Therefore, we conducted the first comparison of PER and OXC as monotherapy in Chinese children and adolescents with FE with or without FBTCSs.

According to the non-inferiority trials of Baulac et al. (2017) and Trinka et al. (2018), the primary outcome was the proportion of patients remaining seizure-free for consecutive months after stabilization at the last evaluated dose. The primary outcome of the present study was the seizure freedom rate at 6 months after the first dose, rather than that for consecutive 6 months, and the difference was attributed to the fact that some of the data were collected retrospectively in our study.

Comparing the results of one phase Ⅲ and two phase Ⅳ clinical trials that evaluated PER treatment for newly diagnosed FE patients (Gil-Nagel et al., 2018; Yamamoto et al., 2020; Wechsler et al., 2022), the seizure freedom rate at 6 months ranged from 33.3% to 74.0%. In a retrospective study by Chinvarun (2022), the seizure freedom rate for newly diagnosed FE with or without FBTCSs at 3, 6, and 12months was 78%, 80%, and 76%, respectively. Previous results showed that seizure freedom was observed in approximately 60% of newly diagnosed patients receiving PER (Yamamoto et al., 2022), which suggested the good effectiveness of PER treatment as monotherapy. In studies reported by Li et al. (2022), Marcohon et al. (2021), and Heyman et al. (2017), 9/83, 5/65, and 2/24 children received PER monotherapy, respectively. The seizure freedom rates in these studies were slightly higher, ranging from 50% to 100%. However, the sample size in the aforementioned studies was small. In our study, the seizure freedom rates in the PER group at 3, 6, and 12 months were 77.1% (54/70), 78.1% (50/64), and 82.9% (29/35), respectively, which were similar to those in the previous studies. At 6 months, 78.2% of patients in the OXC group were seizure-free, which was comparable to the observed 75.9% seizure freedom rate in the previous trial (Zhu et al., 2022). It was also observed that the seizure freedom rate of the PER group slightly increased with time, though not significantly different, compared with the OXC group at each observation point. One explanation could be that children and adolescents who achieved seizure freedom in early treatment periods could sustain longer seizure freedom under PER monotherapy, which was supported by the long-term outcome of FREEDOM Study 342 (Yamamoto et al., 2020). In addition, some epileptic symptoms were considered self-limited, such as BECT. However, these enrolled patients had at least two unprovoked seizures, separated by 24 h in the previous 3 months, which indicated that the interval between seizures was short. Based on experience, the use of ASMs is beneficial in achieving long-term seizure freedom. The proportion of BECT in the PER group was higher than that in the OXC group (32.9% vs. 28.1%), which might contribute to the overall high seizure-freedom rate.

The current study is the first trial comparing the seizure freedom rates between OXC and PER monotherapy. For the PP population, using the 6-month seizure freedom rate as the primary outcome measure, PER monotherapy was non-inferior to OXC in the treatment of children with focal seizures (with/without FBTCSs). The lower limit of the 95% CI in the PER group was above the non-inferiority limit set (62.4%, 80% of the 6-month seizure freedom rate in the OXC group). However, based on baseline characteristics, there were differences in seizure duration and baseline seizure frequency between both groups, which might affect the results. Therefore, we used the Mantel–Haenszel method to evaluate its effect and finally found that the differences would not affect the non-inferiority outcome (risk difference −0.0273, 95% CI: -0.1803 - 0.1257). We ascribed the differences to the selection bias of clinicians who were inclined to treat children and adolescents with a higher seizure frequency with OXC. Patients with certain epilepsies, such as frontal lobe epilepsy, had more frequent seizures during baseline (5–13 seizures per month), and the proportion of patients with FLE in the OXC group was higher than that in the PER group (7.0% vs. 0.0%). Children and adolescents who experienced higher seizure frequency were often treated with ASMs at the first visit, which may explain the shorter seizure duration in the OXC group. Overall, this non-inferiority result was thought to be impressive since OXC had been used as the first-line ASM for the treatment of focal epilepsy (Kanner and Bicchi, 2022).

The PER retention rate in our study was slightly higher than that in the previous studies (Gil-Nagel et al., 2018; Yamamoto et al., 2020; Chinvarun, 2022; Wechsler et al., 2022). This could be explained by the fact that all patients were followed up regularly over the entire study to maximize adherence and that an individualized dosing strategy was implemented to improve tolerability. Consistently, higher retention rates at 3 and 12 months were observed in children and adolescents treated with PER compared with OXC. This might be explained by the higher incidence of AEs (28.1% vs. 21.1%) and AEs leading to dose discontinuation (5.3% vs. 2.9%) in the OXC group. In our study, the estimation using the Kaplan–Meier method on time to treatment failure and seizure freedom was based on the previous clinical trials (Kim et al., 2017; Yamamoto et al., 2020), which aimed to assess the stability of drug efficacy over time. The probability of being seizure-free for PER was 83.5% in our study, which was similar with previous study (Yamamoto et al., 2020). However, the rate of seizure freedom appeared to be higher and treatment failure was lower in the PER group at the later stage of treatment. The explanation could be that, compared with the OXC group, more children and adolescents in the PER group were prospectively followed in an individualized manner, which might improve patients’ treatment compliance and outcomes.

Both drugs were well tolerated, and there were no serious AEs in the two groups. The incidence of AEs with PER was numerically lower than OXC (21.1% vs. 28.1%). However, the incidence of AEs resulting from dose reduction with PER was higher than OXC (8.6% vs. 1.8%). In the PER group, a total of 15 patients (21.1%) experienced at least one adverse event, of which dizziness was the most common, followed by somnolence and irritability. The other three pediatric clinical trials also reported dizziness, somnolence, and irritability, causing treatment discontinuation (Lin et al., 2018; Hwang et al., 2020; Piña-Garza et al., 2020). Moreover, previous studies on PER monotherapy showed that dizziness was the most common AE, which was reported in ≥5% patients (Heyman et al., 2017; Yamamoto et al., 2020; Yamamoto et al., 2022). Notably, three of the patients in the PER group experienced irritability in the current study. Several concerns have been recently raised regarding behavioral and mood-related AEs of PER, which have also caused a high incidence of withdrawal of PER treatment. The first real-world trial in Asia found the most common AEs were irritability and skin rash (Lin et al., 2018). These AEs tended to occur during the period of treatment initiation or at a higher PER dosage. The incidence of skin rash in the PER group was lower than in the OXC group (0% vs. 8.8%, *p* = 0.039). The use of aromatic ASMs is more frequently associated with cutaneous eruption (Błaszczyk et al., 2015).

There were also some limitations in this study. This is a non-randomized, single-center study. The sample size is relatively small. Further prospective studies with a randomized controlled design are needed. In addition, cognitive aspects on which ASMs may have had an effect were not considered. For example, studies have demonstrated that levetiracetam might lead to slight improvements in executive function (Operto et al., 2020a). Another study found that adjunctive PER may improve executive functions (Fernandes et al., 2021). In our study, this aspect had not been explored. However, this is the first study comparing PER and OXC as monotherapy, providing additional insights into the use of PER as monotherapy for children and adolescents with newly diagnosed FE with or without FBTCSs. Our study was conducted in a real-world setting, which indicated an acceptable extrapolation of our results to larger populations.

In conclusion, this preliminary study demonstrated similar efficacy and safety of PER and OXC as monotherapy in children with FE with or without FBTCSs in a real-world setting in China. Our findings highlight the potential use of PER as monotherapy in children and adolescents with newly diagnosed FE.

## Data Availability

The raw data supporting the conclusion of this article will be made available by the authors, without undue reservation.
