# Corrigendum: Comparison of the effectiveness and safety of perampanel and oxcarbazepine as monotherapy in children and adolescents with newly diagnosed focal epilepsy

**DOI:** 10.3389/fphar.2023.1295784

**Published:** 2023-09-29

**Authors:** Jia-Qin Yi, Sheng Huang, Miao-Juan Wu, Jie-Hui Ma, Li-Juan Huang, Song Liang, Dan Sun

**Affiliations:** ^1^ Department of Neurology, Wuhan Children’s Hospital of Tongji Medical College, Huazhong University of Science and Technology, Wuhan, China; ^2^ Department of Pediatric Rehabilitation, Hubei the Third People’s Hospital, Wuhan, China

**Keywords:** perampanel, oxcarbazepine, newly diagnosed focal epilepsy, monotherapy, anti-seizure medications

In the published article, there was an error in Figure 2 as published. The 3-month seizure freedom rate in OXC group should be 80.7% in the original figure. The 6-month seizure freedom rate in OXC group should be 43/55. The corrected Figure 2 and its caption appear below.

**FIGURE 2:**
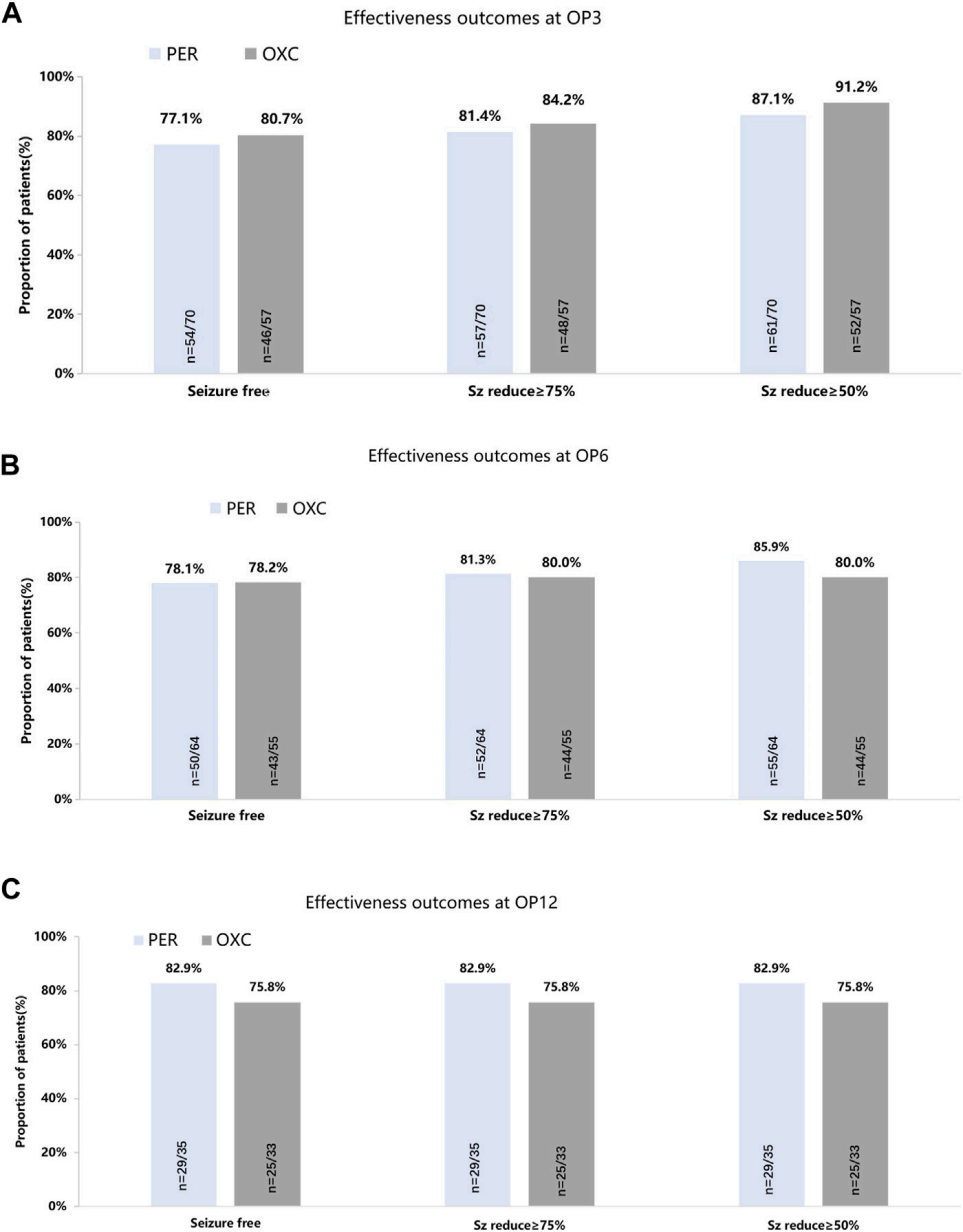
Seizure-response status and seizure-free status on PER/OXC monotherapy. **(A)** OP3; **(B)** OP6; and **(C)** OP12. Seizure free: seizure freedom; Sz reduce ≥75%: seizure reduction ≥75%; Sz reduce ≥50%: seizure reduction ≥50%.

In the published article, there was also an error in the text. The 6-month seizure freedom rate in OXC group should be 43/55.

A correction has been made to **3 Result**, 3.2 Primary endpoint, paragraph 1. This sentence previously stated:

“In the PPS, 78.1% (50/64, 95% CI: 66.0%–87.5%) of children in the PER group and 78.2% (43/56) in the OXC group were seizure-free at 6 months.”

The corrected sentence appears below:

“In the PPS, 78.1% (50/64, 95% CI: 66.0%–87.5%) of children in the PER group and 78.2% (43/55) in the OXC group were seizure-free at 6 months.”

The authors apologize for these errors and state that this does not change the scientific conclusions of the article in any way. The original article has been updated.

